# Sensitivity of Superfolder GFP to Ionic Agents

**DOI:** 10.1371/journal.pone.0110750

**Published:** 2014-10-27

**Authors:** Olesya V. Stepanenko, Olga V. Stepanenko, Irina M. Kuznetsova, Vladislav V. Verkhusha, Konstantin K. Turoverov

**Affiliations:** 1 Laboratory of Structural Dynamics, Stability and Folding of Proteins, Institute of Cytology, Russian Academy of Sciences, St. Petersburg, Russia; 2 Department of Anatomy and Structural Biology, Albert Einstein College of Medicine, Bronx, New York, United States of America; 3 Department of Biochemistry and Developmental Biology, Institute of Biomedicine, University of Helsinki, Helsinki, Finland; 4 Department of Biophysics, St. Petersburg State Polytechnical University, St. Petersburg, Russia; Universidad de Granada, Spain

## Abstract

Superfolder variant of the green fluorescent protein (sfGFP) became a favorite probe for examination of the unfolding–refolding processes of fluorescent proteins with beta-barrel structure owing to its reversible unfolding in comparison with other fluorescent proteins. Its benefit is the proper folding even in fusion constructions with poorly folded polypeptides. We noticed that guanidine thiocyanate affects not only the structure of protein but its chromophore directly. Therefore we studied the influence of ionic denaturants and salts including guanidine thiocyanate, guanidine hydrochloride, sodium chloride and sodium thiocyanate on spectral features of sfGFP. It was shown that moderate amounts of the studied agents do not disrupt sfGFP structure but provoke pronounced alteration of its spectral characteristics. Changes in absorption and CD spectra in visible spectral range indicate the specific binding of SCN^−^ and Cl^−^ anions in the sfGFP chromophore vicinity. The anion binding results in the redistribution of sfGFP molecules with neutral and anionic chromophores. This also hinders the proton transfer in the chromophore excited state, considerably decreasing the fluorescence intensity of sfGFP. Our results indicate that when ionic denaturants are used in the studies of fluorescent protein folding their effect on fluorophore charge state should be taken into account.

## Introduction

Fluorescent proteins (FPs [1]) are an intensively studied large protein family, which is being developed for diverse biological applications [1]–[7]. The alteration and tuning of FP features for desirable practical tasks became possible through the increased knowledge of the relationship between the structural and optical properties of FPs [4], [6]. Some improvements of FPs, however, resulted in side effects, as in the case of the increased sensitivity of yellow FPs (YFPs) to environmental conditions, specifically the pH and the presence of anions [8]. These proteins have found applications in single FP-based biosensors for the detection of surrounding factors, such as pH, ion composition or redox potential inside the cell [9]–[14]. In other cases, a reduced environmental response of FPs would be beneficial [13], [15]. Thus, insight into the influence of surrounding factors on FP features is highly desired.

The effects of simple anions, including halogens, nitrates and thiosulfates, on the spectral features of yellow FPs were recently observed [8], [14], [16]. The binding of these anions around the chromophore is thought to interfere with the formation of the anionic chromophore. In the case of YFP-H148Q, a specific binding site was revealed for iodide located near the chromophore and Gln69 [17]. In the absence of a halide, the center of the cavity adjacent to the chromophore is partially occupied by Gln69, whereas the side-chain of Gln69 is moved out the cavity upon anion binding. In contrast, in the other yellow protein, E2GFP, a specific halide-binding site is located in close contact to the chromophore as revealed by crystallographic data [16]. The halide-binding cavity is composed of Tyr203, Val68, Gln69, Leu42, Val224 and Glu222, with the halide ion H-bonding to some of these residues and a molecule of buried water. During a binding event, the structural changes in the chromophore environment of E2GFP involve the replacement of the water molecule by a halide ion and the extrusion of the O3 atom of the chromophore toward Gln94, thus resulting in the formation of a state termed the open conformation [16]. Several efforts to reduce the anion sensitivity of YFP produced the YFP variants Citrine [13] and Venus [18]. The reduced environmental sensitivity of Citrine, which bears a Gln69Met mutation, was attributed to the tight packing of a Met residue inside the cavity near the chromophore and its inability to undergo the conformational changes that occur for Gln69 of YFP-H148Q [13]. In Venus, a Phe64Leu substitution is considered to be responsible for the weak halide response because the Leu residue precludes ion access to an internal cavity near the chromophore through a series of local conformational changes [15]. Recently, Venus was demonstrated to be strongly sensitive to chloride ions under mildly acidic conditions [19]. There was no specific ion-binding site observed in this case; instead, small structural rearrangements at both lids of the β-barrel cone occurred as a result of the non-specific interaction of ions with the protein.

Our recent studies showed that green FPs can be sensitive to small concentrations of ionic denaturant, such as guanidine thiocyanate (GTC) [20], [21]. The spectral features of a superfolder GFP variant (sfGFP [22]) were significantly affected by the addition of small amounts of GTC, in contrast to the denaturing effects of GTC at elevated concentrations [20]. GTC induces the disruption of the native globular structure of sfGFP at high concentrations (more than 0.9–1.0 M), as indicated by the simultaneous changes in all recorded characteristics, such as the tryptophan fluorescence intensity, parameter *A*, the fluorescence anisotropy, the green chromophore fluorescence intensity and the hydrodynamic dimensions of the protein. However, though low concentrations of GTC produced pronounced changes in the absorption spectrum in the visible range of sfGFP and a drastic decrease in the tryptophan and green chromophore fluorescence of sfGFP (Fig. S1 in File S1, [20]) they caused only subtle changes in the sfGFP structure. As impact of low GTC concentrations is not associated with sfGFP denaturation, unraveling the nature of these effects is of importance for understanding of folding of FPs. Here, we investigated the processes of denaturation of sfGFP in the presence of ionic denaturant guanidine hydrochloride (GdnHCl). We also studied the impact of salts, such as sodium chloride (NaCl) and sodium thiocyanate (NaSCN) on spectral characteristics and structure of sfGFP.

## Materials and Methods

### Gene expression and protein purification

The plasmid pET-28a(+)-sfGFP encoding superfolder GFP [22] with a poly-histidine tag was constructed as described previously [23] and transformed into an *Escherichia coli* BL21(DE3) cells (Invitrogen). The expression of sfGFP was induced by incubating the cells with 0.5 mM isopropyl-beta-D-1-thiogalactopyranoside (IPTG; Fluka, Switzerland) for 24 h at 23°C. Recombinant protein was purified with Ni^+^-agarose packed in His-GraviTrap columns (GE Healthcare, Sweden). The purity of the protein was at least 95%, as indicated by SDS-PAGE with a 15% polyacrylamide gel [24]. The optical density of protein samples did not exceed 0.2. Measurements were performed in 100 mM Na-phosphate-buffered solution at pH 8.0.

### Spectrophotometric experiments

Absorption spectra were recorded using a U-3900H spectrophotometer (Hitachi). The experiments were performed in 101.016-QS microcells (5×5 mm) (Hellma) at room temperature.

### Fluorescence spectroscopy

Fluorescence experiments were conducted using a Cary Eclipse spectrofluorometer with FLR microcells (10×10×4 mm; Agilent Technologies, Australia).

Fluorescence intensity was corrected on the primary inner filter effect according to approach which we recently proposed [25], [26]:



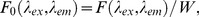
(1)where W is a correction factor calculated as 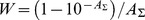. Here 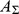 is the total absorbance of the exciting light in the solution [25].

Fluorescence anisotropy and fluorescence lifetime were measured using home-built spectrofluorometers with steady-state and time-resolved excitation [27] using micro-cells (101.016-QS 5×5 mm; Hellma, Germany).

The tryptophan fluorescence of the protein was excited in the long-wave absorption spectrum edge (λ_ex_ = 297 nm), where the contribution of tyrosine residues in the bulk protein fluorescence is negligible. The position and form of the fluorescence spectra were characterized by the parameter *A* =  *I*_320_/*I*_365_, where *I*_320_ and *I*_365_ are the fluorescence intensities at the emission wavelengths of 320 and 365 nm, respectively. The values of parameter *A* and of the fluorescence spectrum were corrected by the instrument sensitivity. The anisotropy of tryptophan fluorescence was calculated by the equation 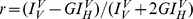, where 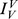 and 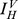 are the vertical and horizontal components of the fluorescence intensity when excited by vertically polarized light, and *G* is the relationship of the vertical and horizontal components of the fluorescence intensity when excited by horizontally polarized light 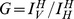, λ_em_ = 365 nm [27]. The specific “green” fluorescence of sfGFP was excited at 390 or 490 nm which correspond to the maxima of the absorbance bands of the neutral and anionic forms of the chromophore. Emission of sfGFP with neutral chromophore was detected at 450 nm and of that with anionic chromophore was recorded at 510 nm.

The protein solutions were prepared by manually mixing concentrated protein solution (50 µl) with a buffer containing the desired concentrations (450 µl) of guanidine thiocyanate (GTC), sodium thiocyanate (NaSCN), guanidine hydrochloride (GdnHCl) or sodium chloride (NaCl). GTC (Sigma-Aldrich, USA), NaSCN (Sigma-Aldrich, USA), GdnHCl (AppliChem, Germany), NaCl (Reachem, Russia) and Na_2_SO_4_ (Sigma-Aldrich, USA) were used without further purification. The concentration of stock solutions of GTC, NaSCN, and GdnHCl were determined by the refraction coefficient with an Abbe refractometer (LOMO, Russia). The impact of GTC/NaSCN/NaCl and GdnHCl on the different fluorescent characteristics of sfGFP was recorded following protein incubation in solutions of the appropriate additive at 23°C for 24 h and 96 h, respectively. The spectrofluorometer was equipped with a thermostat that held the temperature constant at 23°C.

*pH titrations* – pH titrations were performed using a series of buffers: 50 mM NaOAc (Sigma-Aldrich, USA) for pH 4.5, and 50 mM NaH_2_PO_4_ (Sigma-Aldrich, USA) for pH 5.3–9.4. Protein concentration was 5 µM.

### Circular dichroism measurements

CD spectra were obtained using a Jasco-810 spectropolarimeter (Jasco, Japan). The CD spectra in the visible range were scanned from 550 to 320 nm with a step size of 0.1 nm using a 10-mm path length cell. For all spectra, an average of 3 scans was obtained. The CD spectra of the appropriate buffer solutions were recorded and subtracted from the protein spectra.

*The determination of the fractions of anionic and neutral forms of sfGFP at definite concentration of studied ionic agent -* The existence of isosbestic point in the absorption spectra of sfGFP indicates that the absorbance at the wavelength 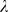 is can be determined as follows:
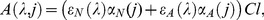
(2)where *C* is the molar protein concentration and *l* is an optical path length, *j* is the concentration of any target agent (GTC, NaSCN or GdnHCl) affecting the redistribution of the fractions of neutral (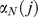) and anionic (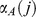) forms of sfGFP (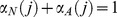); 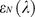 and 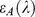 are molar absorptions of these fractions at the given conditions.

The ratio of any two spectra is constant in the wavelength range where only one component absorbs [34]. This allows choosing the wavelength λ_1_ where the impact of neutral form to absorption is negligible. Then the Eq. (2) for this wavelength will be as follows:
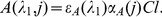
(3)Consequently, the ratio of 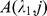 to 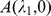, which corresponds to the absence of ionic agents, will give the value of 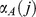 if 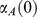 is known.

To determine 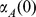 (and 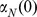) we have made two assumptions: we supposed that molar absorption spectrum of neutral form of sfGFP has a Gaussian form and that the contribution of anionic form at the wavelength λ_2_ which is symmetrical to the isosbestic point λ*_IP_* is negligible. Then:
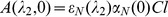
(4)and 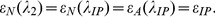
(5)


Furthermore, if one takes into account that the absorption in the isosbestic point can be presented as:
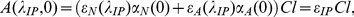
(6)than on the basis of Eq. (4–6) it is easy to obtain:
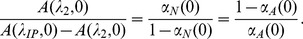
(7)


## Results

The effect of ionic denaturants, such as GTC and GdnHCl, and salts, such as NaSCN and NaCl, on the spectral properties of sfGFP were probed by spectroscopic methods (absorbance, fluorescence, and circular dichroism).

**Figure 1:**
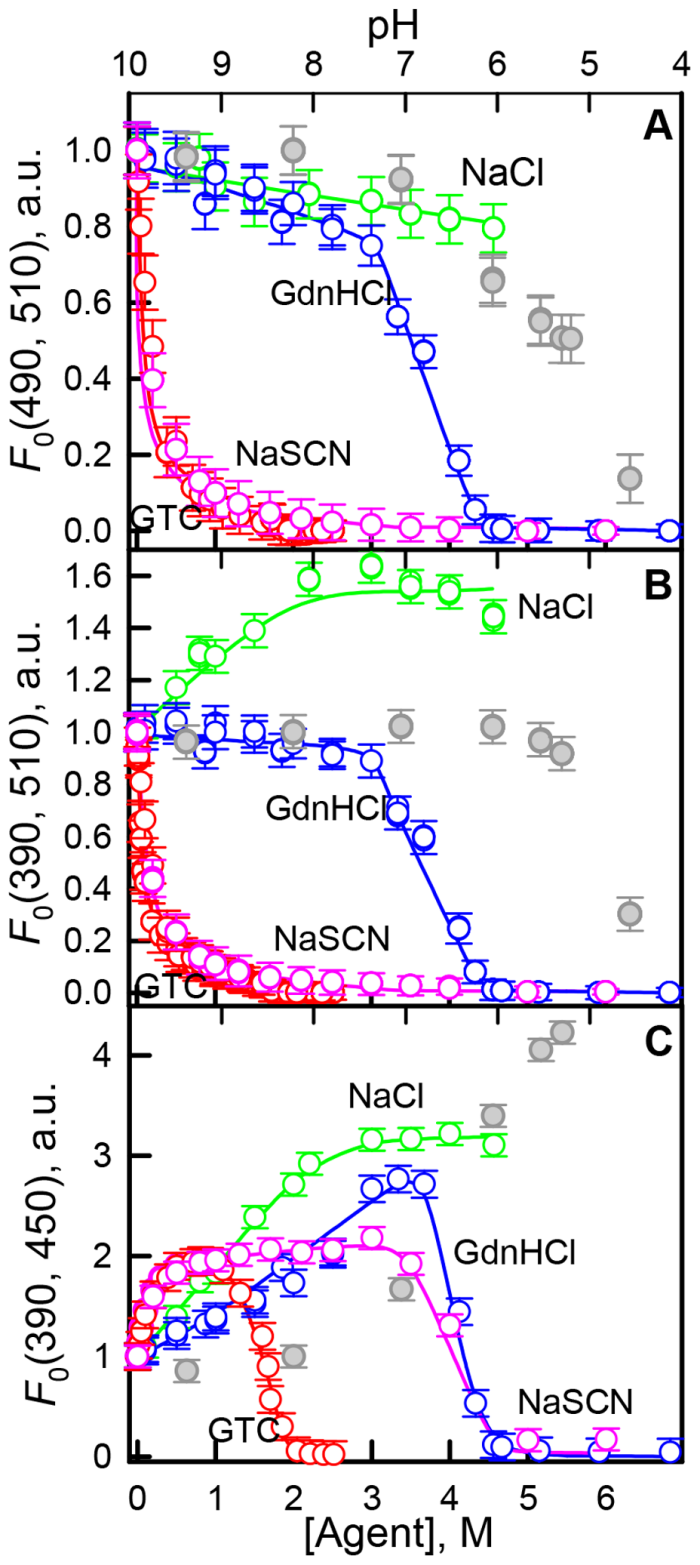
Ionic denaturants and salts effects on fluorescence of anionic and neutral chromophore of sfGFP. Changes in the anionic chromophore fluorescence intensity, λ_ex_ = 490 nm, λ_em_ = 510 (***A***) and λ_ex_ = 390 nm, λ_em_ = 510 (***B***). Changes in direct fluorescence from neutral form, λ_ex_ = 390 nm, λ_em_ = 450 nm (***C***). The value of fluorescence intensity was corrected for the primary inner filter effect (see “Materials and methods” section). Agents used were GTC (red circles) and GdnHCl (blue circles), NaSCN (pink circles) and NaCl (green circles). Data on fluorescence intensity of sfGFP in different pH are shown in gray circles, axis of abscissas for these data is on top.

Figure 1 represents the changes of chromophore fluorescence of sfGFP recorded during denaturation induced by GTC and GdnHCl and in the presence of salts NaSCN and NaCl. The specific “green” fluorescence of sfGFP was excited at 390 (490) nm where the neutral and anionic forms of the chromophore in sfGFP absorb. It is known that the excitation of the neutral chromophore is followed by its deprotonation with the formation of the excited state of anionic chromophore I^*^. Emission from this state results in fluorescence spectrum similar to that obtained at direct excitation of anionic chromophore at 490 nm (Fig. S2 in File S1). Direct emission of the neutral form of the chromophore with low quantum yield is still possible [28], [29] and can be detected as a shoulder at approximately 450 nm in the fluorescence spectra of sfGFP at excitation wavelength of 390 nm (Fig. S2 in File S1, second column). Thus, the fluorescence intensity emitted by the chromophore in the neutral form was detected at 450 nm and anionic chromophore was recorded at 510 nm. The value of fluorescence intensity was corrected for the primary inner filter effect (see “Materials and methods” section).

The addition of GTC and GdnHCl results in both anionic and neutral chromophore fluorescence change (Fig. 1) at the range of denaturants concentration where protein unfolding does not yet occur (Fig. S3 in File S1). The GTC-induced alterations of sfGFP fluorescence are more pronounced as compared to GdnHCl action. NaCl and NaSCN salts provoke the changes of anionic and neutral chromophore fluorescence as well (Fig. 1), which is not associated with any structural perturbations (Fig. S3 in File S1). Small concentrations of NaSCN salt causes the drop (rise) of anionic (neutral) chromophore fluorescence to the same extent as GTC does (Fig. 1, *A* and *B*).

At low denaturant concentrations, an increase in the GTC concentration is accompanied by a sharp and significant decrease in the value of tryptophan fluorescence and green chromophore fluorescence at the excitation and absorption bands of the neutral and anionic chromophores (Figs. S2 in File S1). The change of sfGFP tryptophan fluorescence produced by a low amount of GTC is also observed after the addition of all other agents, but GdnHCl has a noticeably diminished effect compared to the other agents (Fig. S2 in File S1).

**Figure 2:**
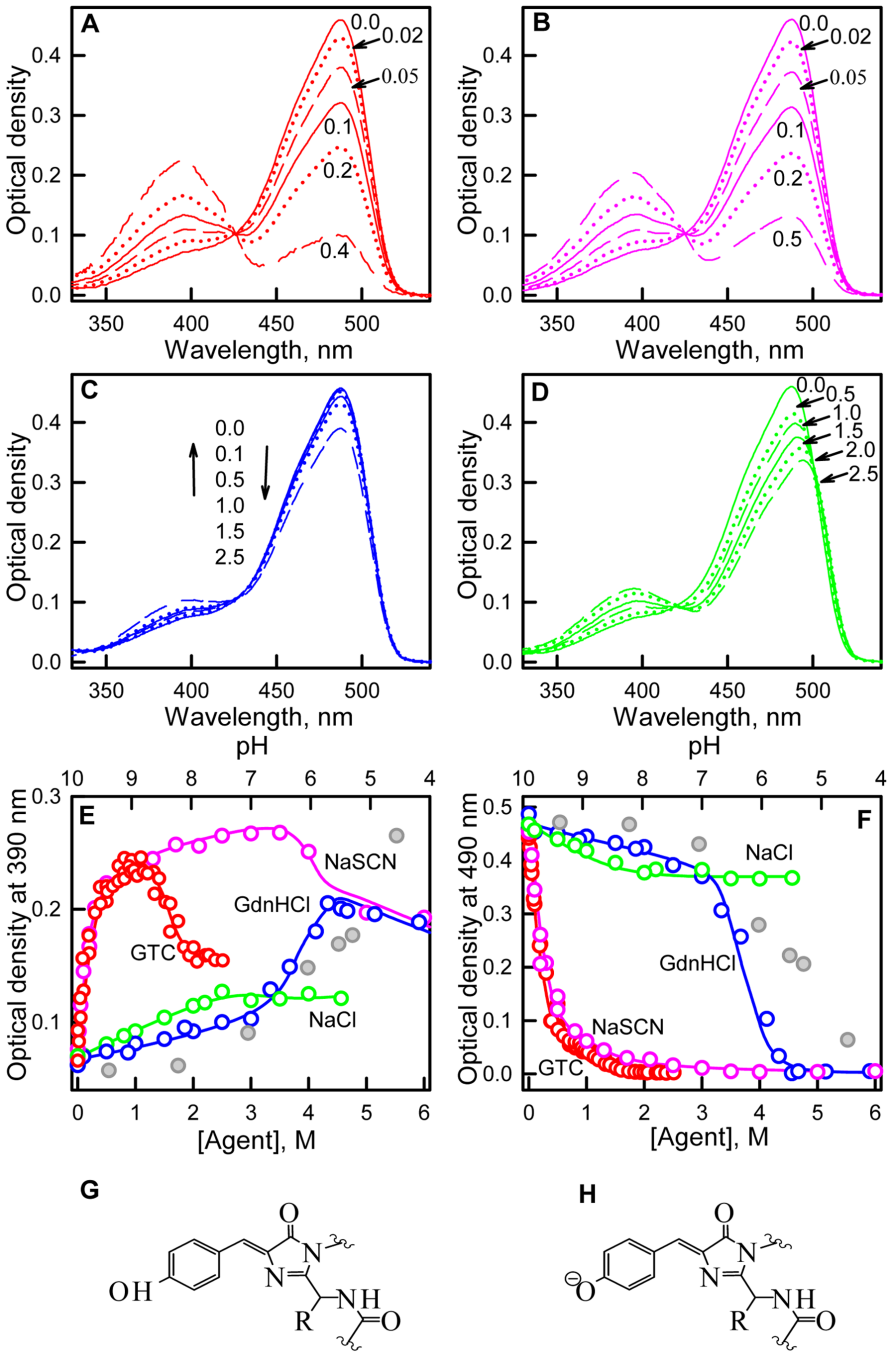
Ionic denaturants and salts effects on absorption of neutral and anionic chromophore forms of sfGFP. (***A***) Absorption spectra of sfGFP in the buffered solution and in the presence of 0.02, 0.05, 0.1, 0.2 and 0.4 M GTC. (***B***) Absorption spectra of sfGFP in the buffered solution and in the presence of 0.02, 0.05, 0.1, 0.2 and 0.5 M NaSCN (***C***) Absorption spectra of sfGFP in the buffered solution and in the presence of 0.1, 0.5, 1.0, 1.5 and 2.5 M GdnHCl. (***D***) Absorption spectra of sfGFP in the buffered solution and in the presence of 0.5, 1.0, 1.5, 2.0 and 2.5 M NaCl (***E***) Changes in optical density at the absorption maxima for the neutral chromophore. (***F***) Changes in optical density at the absorption maxima for the anionic chromophore. Agents used included ionic denaturants, such as GTC (red circles) and GdnHCl (blue circles), and salts, such as NaSCN (pink circles) and NaCl (green circles). Data on absorption of neutral and anionic chromophore of sfGFP in different pH are shown in gray circles, axis of abscissas for these data is on top. Schematic representation of neutral (***G***) and anionic (***H***) chromophores is given.

We detected that GTC, GdnHCl in pre-denaturing concentrations and NaCl and NaSCN salts induce significant alterations of the visible absorption of sfGFP (Fig. 2). The absorption spectrum in the visible range of sfGFP in the buffered solution exhibits a maximum at approximately 490 nm and a shoulder at 390 nm (Fig. 2). These two peaks in the absorption spectra in the visible range of sfGFP are attributable to the anionic and neutral forms of the chromophore (Fig. 2, *G* and *H*) [8], [30]–[32]. GTC at pre-denaturing concentrations substantially affects the absorption spectrum in the visible range of sfGFP (Fig. 2). A reduction in the absorption band at approximately 490 nm, which corresponds to the anionic form of the green chromophore, is accompanied by an increase in the absorption band at 390 nm, which corresponds to the neutral form of the green chromophore (Fig. 2, *E* and *F*). Notably, the optical density at approximately 425 nm remains unaltered in these GTC concentrations, which is an isosbestic point where molar absorption of the neutral and anionic chromophore forms of sfGFP is the same (Fig. 2).

The curves of both optical densities at 390 and 490 nm in the presence of NaSCN concentrations up to 0.5 M are almost coincident to those observed in the same range of GTC concentrations (Fig. 2). The addition of NaCl to sfGFP solution also results in some increase in absorbance at 390 nm and a decrease in absorbance at 490 nm (Fig. 2). GdnHCl at concentrations up to 2.5 M slightly affect the sfGFP absorption spectra in the visible region (Fig. 2). In the absorption spectra in the visible range of sfGFP in the presence of GdnHCl and NaSCN the same isosbestic point at 425 nm is also observed. In the presence of NaCl, the position of the absorption band of the neutral chromophore remains unaltered and the absorption band of the anionic chromophore is red-shifted to 494 nm. This results in appearance of the second isosbestic point at absorption spectra of sfGFP at 500 nm in addition to isosbestic point at 420 nm (Fig. 2).

**Figure 3:**
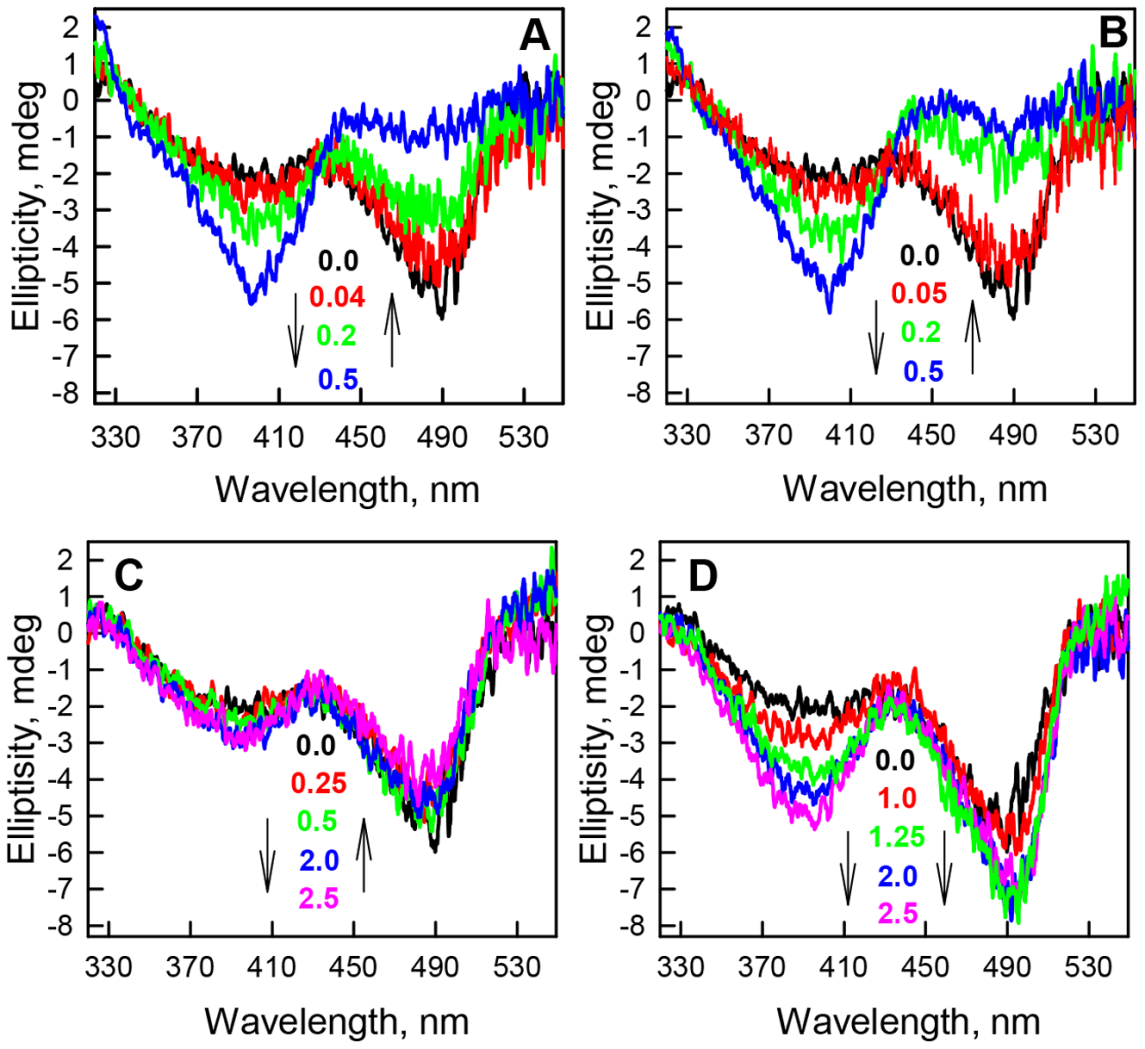
The effect of ionic denaturants and salts on the CD in the visible range of sfGFP. (***A***) CD spectra of sfGFP in the visible-UV region were recorded at final GTC concentrations of 0.0 (black line), 0.04 (red line), 0.2 (green line), and 0.5 M (blue line). (***B***) CD spectra of sfGFP in the visible-UV region were recorded at final NaSCN concentrations of 0.0 (black line), 0.05 (red line), 0.2 (green line), and 0.5 M (blue line). (***C***) CD spectra of sfGFP in the visible-UV region were recorded at final GdnHCl concentrations of 0.0 (black line), 0.25 (red line), 0.5 (green line), 2.0 M (blue line) and 2.5 M (pink line). (***D***) CD spectra of sfGFP in the visible-UV region were recorded at final NaCl concentrations of 0.0 (black line), 1.0 (red line), 1.25 (green line), 2.0 M (blue line) and 2.5 M (pink line).

The effects of the studied agents on the chromophore environment were tested by CD in the visible range. The CD spectrum in the visible range of sfGFP in the absence of denaturant has a clearly distinguishable negative band at 490 nm and a negative shoulder at 390 nm (Fig. 3, *A*, black line). As the GTC concentration increases, a swapping of the intensities of these two bands is observed, with an increase in band intensity at 390 nm and a concomitant decrease in band intensity at 490 nm (Fig. 3, *A*). The ratio of the bands' intensities in the CD spectra in the visible range of sfGFP at 0.5–0.8 M GTC is inverted from the ratio in the absence of the denaturant, such that the more sizable band at 390 nm exceeds the intensity of the band at 490 nm (Fig. 3, *A*). The addition of NaSCN similarly affects the CD spectra in the visible range of sfGFP, resulting in an increase in band intensity at 390 nm and a decrease in band intensity at 490 nm (Fig. 3, *B*). The extent of these changes is equal to that observed in the presence of GTC. GdnHCl similarly affects the CD spectra in the visible range of sfGFP, but the magnitude of the changes in the bands is extremely low (Fig. 3, *C*). The CD spectra in the visible range of sfGFP in the presence of NaSCN, GTC and GdnHCl contain an isosbestic point between bands of neutral and anionic form of chromophore. NaSCN and GTC have similar and pronounced effect on the CD spectra of sfGFP. The change of the CD spectra in the visible range of sfGFP in the presence of GdnHCl at concentrations up to 2.5 M is negligible. In contrast, NaCl induces the increase in the intensities of both bands at 390 and 490 nm and as result the CD spectra in the visible range of sfGFP in the presence of NaCl do not have any isosbestic point (Fig. 3, *D*).

### The influence of ionic agents on the proportion between anionic and neutral forms of sfGFP

**Figure 4:**
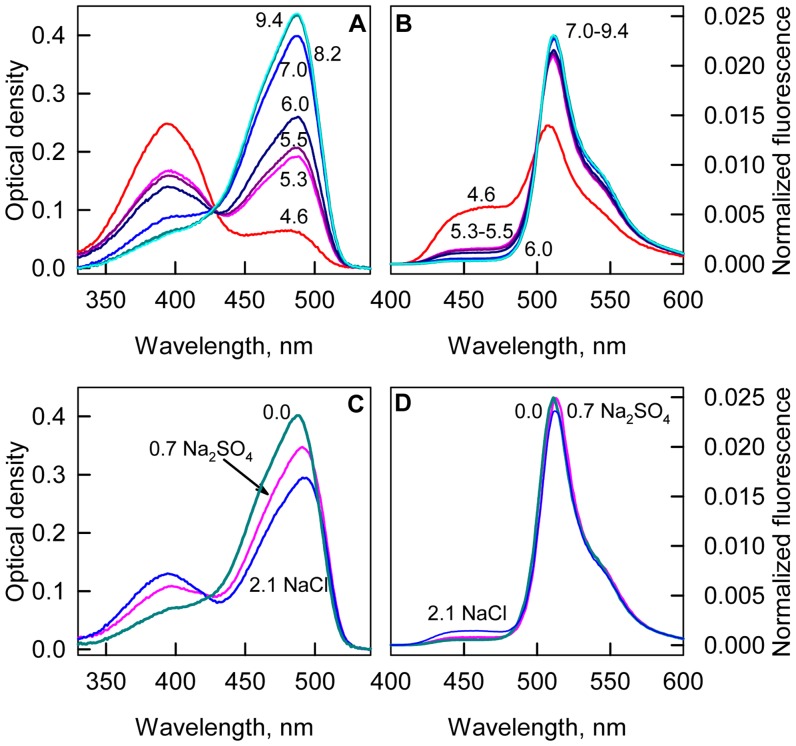
The effect of pH and ionic strength on sfGFP spectroscopic characteristics. Absorption spectra (***A***) and fluorescence spectra (***B***) of sfGFP in the solutions with different pH. Fluorescence was excited at 390 nm and normalized to total fluorescence at current pH value. The numbers on panels A and B are the values of pH in solution. Absorption spectra (***C***) and fluorescence spectra (***D***) of sfGFP in the buffered solution and in solutions with the equal ionic strength but containing 0.7 M Na_2_SO_4_ or 2.1 M NaCl. Fluorescence was excited at 390 nm and normalized in the same manner as data on panel B.

In sfGFP solutions, molecules with an anionic chromophore are dominant, and it forms the absorption spectrum with a major peak at 490 nm and a shoulder at 390 nm (Fig. 2). The addition of any studied agent in moderate concentrations alters the proportion of molecules with neutral and anionic chromophores as can be observed from the increase in the absorption band of the neutral chromophore and the decrease in the absorption band of the anionic chromophore (Fig. 2). An isosbestic point in the absorption spectra of sfGFP in the presence of GTC, NaSCN and GdnHCl indicates that only two types of molecules exist in the presence of moderate concentrations of these agents, and the observed alterations of the absorption spectra in the visible range are caused only by changes in the proportion between neutral and anionic chromophore rather than by a change of the absorbance of the sfGFP molecules holding neutral and anionic chromophores. The fact that equilibrium exists between neutral and anionic form is further confirmed by pH-titration of sfGFP (Fig. 4, *A* and *B*), that yields the same value of isosbestic point in absorption spectra as in our experiment on influence of ionic denaturants GTC and GdnHCl and NaSCN salt on sfGFP characteristic. Normalized fluorescence spectra of sfGFP chromophore (λ_ex_ = 390 nm) in different pH also have isosbestic point (Fig. 4, *B*) and are characterized by visible increase of direct fluorescence of neutral chromophore with concomitant decrease of anionic chromophore fluorescence.

It is worth to note that response of sfGFP to different agent is not equivalent: redistribution of sfGFP molecules with neutral and anionic chromophore is most pronounced in the presence of GTC and NaSCN, less profound in NaCl, and neglected in GdnHCl (Fig. 2, *C* and *D*). This argues for a specific influence of studied agents in addition to non-specific due to ionic strength change. To confirm this we studied the characteristics of sfGFP in solutions with equal ionic strength containing 0.7 M Na_2_SO_4_ and 2.1 M NaCl (Fig. 4, *C* and *D*). It is evident that in solution containing NaCl the equilibrium between neutral and anionic forms of sfGFP shifts more than in solution containing Na_2_SO_4_. The presence of NaCl induces higher effect on sfGFP fluorescence then Na_2_SO_4_ does. These data argue for specific action on sfGFP of NaCl salt.

The behavior of absorption spectra in the visible range of sfGFP in the presence of NaCl is more complex (Fig. 2). The two isosbestic points indicate the existence of three types of sfGFP molecules with different absorption spectra, with longwave and shortwave bands not sufficiently overlapping with each other (otherwise there would be a second isosbestic point). Evidently, the first two types of sfGFP molecules represent the protein bearing the neutral and anionic chromophore. The absorption spectrum of the third type of sfGFP molecule is apparently red-shifted with respect to the absorption spectrum of the anionic chromophore of sfGFP. This reflects that the third type of sfGFP molecule contains the anionic chromophore which excited state is stabilized [30], [32].

### Determination of molar absorption spectra of neutral and anionic forms of sfGFP

**Figure 5:**
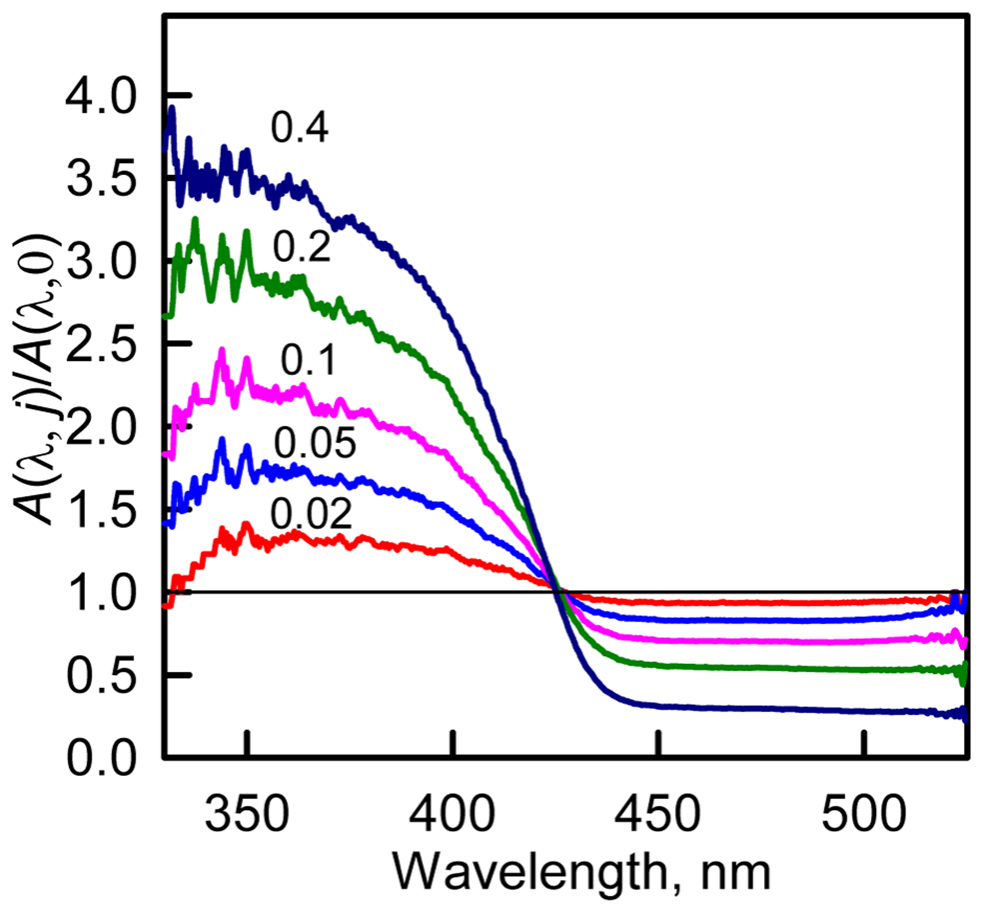
The change of the ratio of 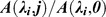. The dependences calculated on the base of absorption spectra of sfGFP in the buffered solution and in the presence of 0.02, 0.05, 0.1, 0.2 and 0.4 M GTC are shown.

We used the absorption spectra in the visible range of sfGFP in the presence of GTC, NaSCN and GdnHCl to extract absorption spectra of neutral and anionic chromophore according to the equation (2). The fractions of anionic and neutral forms of sfGFP at definite concentration of studied ionic agent were determined as described in “Materials and methods” section. To define the wavelengths λ_1_ where the impact of neutral form to absorption is negligible we calculated the ratio 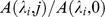 (Fig. 5). This ratio should be constant in the wavelength range where only one component absorbs [34]. On the basis of the plots presented in Figure 5 the wavelengths λ_1_ was chosen to be equal to 490 nm. According to the assumption on a Gaussian form of absorption spectrum of neutral form of sfGFP we assigned the wavelengths λ_2_ as 355 nm which is symmetrical to the isosbestic point λ*_IP_* at 425 nm. The values of 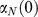 and 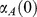 calculated according to the equation (7) for sfGFP in buffered solution are 0.22 and 0.78. The value of neutral chromophore for sfGFP in buffered solution is in good agreement with the value of 0.18 for dark fraction in sfGFP determined by FCS by Cotlet [35]. Thus, on the basis of 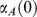
and experimentally determined absorbance at 490 nm the fraction 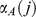 and consequently 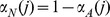 can be calculated.

**Figure 6:**
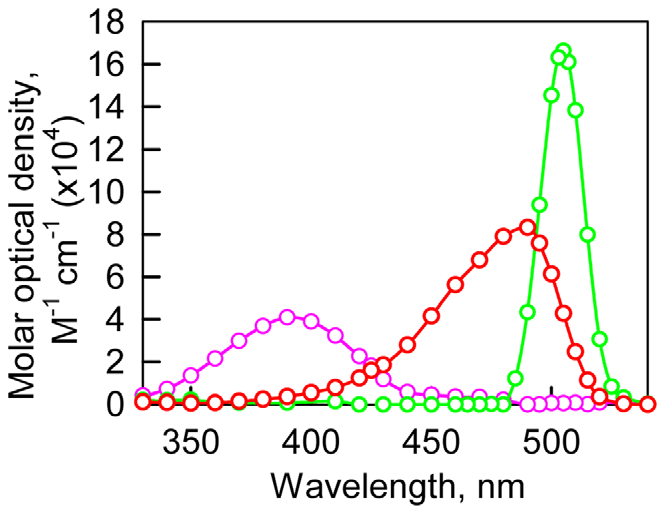
Molar absorption spectra of neutral (pink), anionic (red) and complexed with Cl^−^ (green) states of sfGFP. Spectra decomposition was made on the basis of absorption spectra of sfGFP in the presence of studied agents. For details see the discussion section.

Using calculated fraction of neutral and anionic chromophore in different agents we restored the molar absorption spectra of neutral and anionic chromophore by non-linear regression using SigmaPlot software (Fig. 6). The molar absorption spectra of neutral and anionic form calculated for sfGFP are in good agreement with molar absorption spectra of protonated state (A′-state) and an anionic state (M-state) obtained for EGFP [36].

The molar absorption spectra of neutral and anionic chromophore were used to calculate the molar absorption spectrum of the third type of sfGFP molecule on the basis of the absorption spectra in the visible range of sfGFP in the presence of NaCl (Fig. 6). As the addition of all studied agents inhibits the anionic chromophore of sfGFP we suppose that their specific action is consequence of binding of negatively charged ions of Cl^−^ and SCN^−^. In this case we can suppose that third type of sfGFP molecules which are observed at NaCl action represents the protein containing the anionic chromophore with bound Cl^−^ ion. It is likely, that Cl^−^ binding alters the local environment of the chromophore and results in slight changes in the distances between the chromophore and surrounding residues that can stabilize the chromophore excited state. As was shown previously for avGFP [30], its chromophore excited state is stabilized through an electrostatic attraction between the chromophore's carbonyl oxygen and the positively charged side chain of Arg96. Moreover, opposite effect of GTC/NaSCN and NaCl on the CD spectra in the visible range of sfGFP (Fig. 3) can witness that the thiocyanate (SCN^−^) and Cl^−^ anions have diverse binding sites in the chromophore environment. The diverse binding sites for SCN^−^ and Cl^−^ anions probably explain that we do not observe the third state in the presence of NaSCN and GTC spectroscopically.

## Discussion

Mutant form sfGFP which was developed to overcome some drawbacks of other green FPs [33] demonstrates strong folding efficiency and a low tendency toward aggregation [22]. The study of the processes of folding – unfolding of sfGFP induced by GTC revealed that this protein is sensitive to low GTC concentrations (Fig. S1 in File S1). Here we showed that other ionic denaturant GdnHCl and salts, such as NaSCN and NaCl, also affect sfGFP's features.

### The effect of studied agents on sfGFP fluorescence

**Figure 7:**
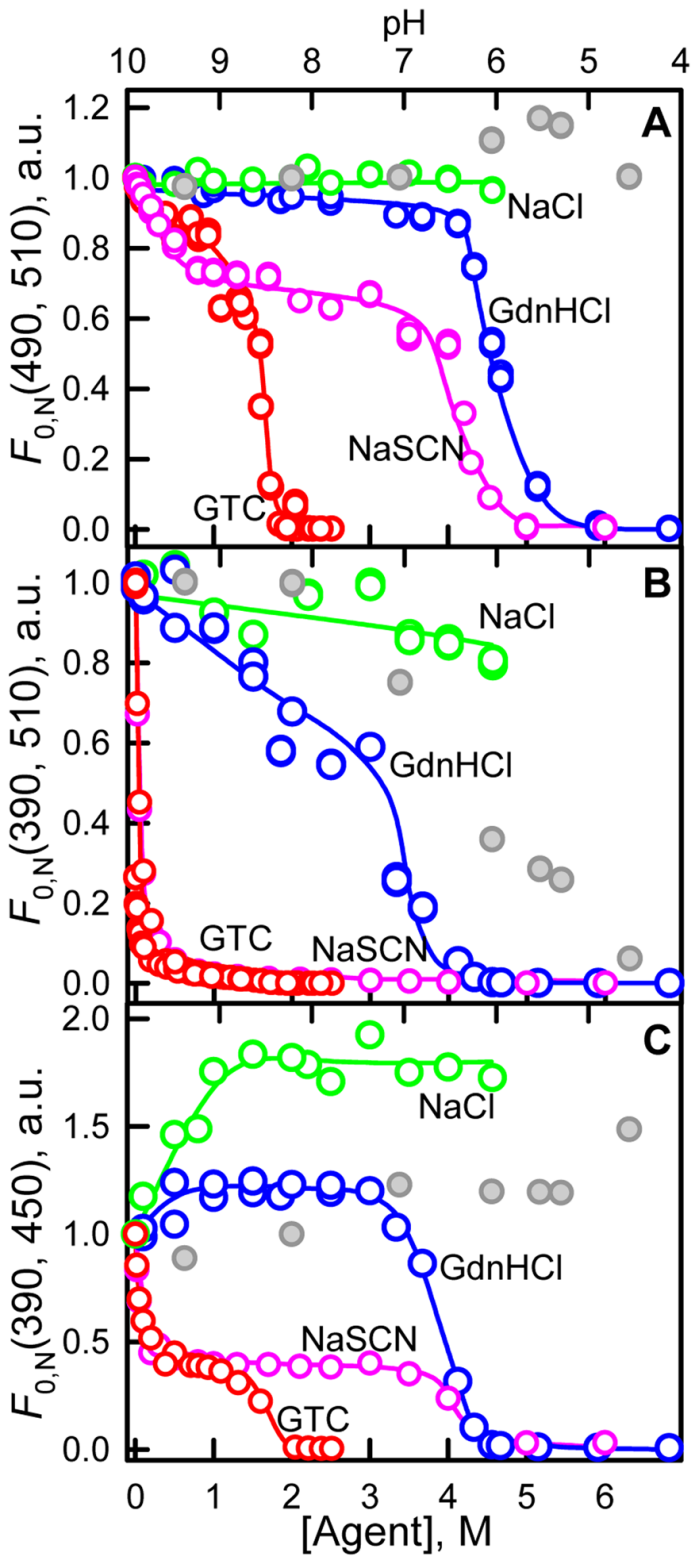
Ionic denaturants and salts effects on fluorescence of anionic and neutral chromophore of sfGFP. (***A***) Changes in the chromophore fluorescence intensity upon excitation of anionic chromophore at wavelength of 490 nm and detection at 510 nm. (***B***) Changes in the chromophore fluorescence intensity upon excitation of neutral chromophore at wavelength of 390 nm and detection at 510 nm. (***C***) Changes in direct fluorescence from neutral form at 450 nm at excitation wavelength of 390 nm. The value of fluorescence intensity corrected for the primary inner filter effect was normalized to equal chromophore concentration (see “Discussion” section). Agents used were GTC (red circles) and GdnHCl (blue circles), NaSCN (pink circles) and NaCl (green circles). Data on fluorescence intensity of sfGFP in different pH are shown in gray circles, axis of abscissas for these data is on top.

As the addition of all studied agent shifts the equilibrium between the neutral and anionic chromophore before analyzing the effects of different agent on chromophore fluorescence we must normalize fluorescence intensity, which was corrected for inner filter effect, to equal chromophore concentration:
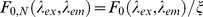
here 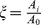, where *A_0_* and 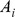 are absorbance of solution in the absence of any agent and in the presence of target agent. After normalization the plots presented in Figure 1 are transformed to the plots presented in Figure 7.

#### The fluorescence of anionic chromophore (λ_em_ = 510 nm) at excitation at 490 nm

We should notice that sharp decrease of anionic chromophore fluorescence at addition of small GTC concentrations makes the denaturing transition indistinguishable on dependencies of the chromophore fluorescence intensity. Denaturing transition at 0.9–2.0 M GTC can be observed on the dependencies of parameter *A* and fluorescence anisotropy of sfGFP (Fig. S3 in File S1). The normalization of fluorescence intensity of anionic chromophore (λ_em_ = 510 nm at λ_ex_ = 490 nm) of sfGFP allows to linearize the fluorescence dependences, as result denaturing transitions become clearly seen (Fig. 7, *A*).

The NaSCN salt was shown to exert denaturing action on protein structure as it is evident from the changes of fluorescence intensity of anionic chromophore of sfGFP (Fig. 7, *A*), and confirmed by dependences of parameter *A* and fluorescence anisotropy (Fig. S3 in File S1). It is worth to note that the denaturing transition occurs at 3.0–5.0 M NaSCN that is close to GdnHCl-induced denaturation transition (3.3–4.5 M, Fig. S3 in File S1). Still incubation of sfGFP at high NaSCN concentrations does not led to complete sfGFP unfolding which is indicated by higher value of parameter *A* and fluorescence anisotropy of sfGFP in these conditions as compared to fully denatured protein (Fig. S3 in File S1). Apparently, NaSCN is weaker denaturant in comparison with GdnHCl. According to these data we can assume that strong denaturing action of GTC is cased by additive action of guanidine and thiocyanate components.

The normalized fluorescence intensity of anionic chromophore does not changed in the response to NaCl, moderate concentrations of GdnHCl, and in the rage of pH from 4.5 to 9.4. This is an expected result showing that all factors affecting the fluorescence of anionic chromophore are considered. At pre-denaturing concentrations of GTC and NaSCN a small decrease of fluorescence intensity of anionic chromophore is preserved. Probably, GTC and NaSCN exert a quenching action on the anionic chromophore's fluorescence. This quenching effect is most likely due to the sulfur atom of the SCN^−^ anion when it is present near the chromophore.

#### The fluorescence of anionic chromophore (λ_em_ = 510 nm) at excitation at 390 nm

This normalization procedure does not align the fluorescence intensity of anionic chromophore excited at absorption of neutral chromophore (λ_em_ = 510 nm at λ_ex_ = 390 nm) of sfGFP. This dependences display a noticeable decrease in the presence of NaCl and moderate concentrations of GdnHCl and sharp decrease in the presence of low concentrations of GTC and NaSCN. It is well-known that neutral chromophore undergoes deprotonation under excitation. Fully deprotonated anionic exited state I^*^ is preceded by the excited-state intermediate I_0_^*^ [37]. The intermediate I_0_^*^ is characterized by a partial protonation of Glu222 and a shift of protons of the hydrogen bond network in which involved Ser65 and Tyr66 residues of the chromophore, and a water molecule, and the Ser205 and Glu222 residues of near environment in avGFP. The presence of a Cl^−^ or SCN^−^ anion near the chromophore may interrupt the formation of the intermediate state I_0_^*^ by restricting the wagging motion of the chromophore, which was shown to be necessary for finding the proper conformation of the chromophore and its immediate environment to enable proton transfer with a low energetic barrier [38]. This means that normalization procedure of *F*_0_(510, 390) is more complex as it should take into account the inhibition of proton transfer in the chromophore excited state. The reasons of noticeable decrease of *F*_0, *N*_ (510, 390) in the pre-denaturing concentrations of GdnHCl is not clear. Previously it was shown that positively charged ions of guanidine GdnH^+^ can greatly affect the features of proteins, e.g. creatine kinase [39], actin [40], [41], carboanhydrase [41], [42], and ligand-binding protein [43]–[45]. The GdnH^+^ cations are able to interact with the protein surface under appropriate conditions. Notably, proteins that have an isoelectric point at acidic pH have a negative charge at neutral pH. In this case, positively charged GdnH^+^ ions were shown to neutralize the negative charge of the protein through interaction with the carboxyl groups of glutamic and aspartic amino acids and the amide groups of glutamine and asparagine that are present on the protein's surface. The isoelectric point of sfGFP is 6 [46], which means that the protein surface bears a negative charge under neutral conditions. Thus GdnH^+^ cations can bind with sfGFP and induce local structural perturbations in sfGFP that result in alteration of the protein fluorescence. Moreover, the bound GdnH^+^ ions can preclude the access of Cl^−^ anions to the chromophore cavity of the sfGFP that might explain the lesser response of sfGFP to GdnHCl then to NaCl.

#### The fluorescence of neutral chromophore (λ_em_ = 450 nm) at excitation at 390 nm

The interpretation of fluorescence intensity of neutral chromophore (λ_em_ = 450 nm at λ_ex_ = 390 nm, Fig. 7, *C*) is even more complicated as here the secondary inner filter effect is present. This effect is caused, firstly, by re-absorption of fluorescence light due to overlap of long wavelength absorption band (absorption of anionic chromophore) and the fluorescence spectrum (direct emission of neutral chromophore), and, secondly, by re-absorption of the tryptophan emission by neutral chromophore. As we can see, the quenching of sfGFP tryptophan fluorescence is present in all studied agents (Fig. S2 in File S1). The reason of the tryptophan fluorescence quenching can be not only because of re-absorption but because of increased non-radiative energy transfer from the tryptophan residue of sfGFP to the chromophore in its neutral form.

In summary, we have shown that salts and a set of denaturants widely used for study the protein folding may exert the specific action on sfGFP spectral characteristics due to the anion binding in the chromophore vicinity. In the case of GTC and NaSCN, which was shown to be a week denaturant, it is SCN^−^ anions. In the case of NaCl, it is Cl^−^ anions. In the case of GdnHCl, the binding of positively charged GdnH^+^ ions to the surface of sfGFP under neutral conditions diminishes the effect of Cl^−^ anions on the chromophore of sfGFP. The anion binding affects the proton wire around the chromophore of sfGFP thus changing equilibrium to the neutral form of the chromophore and quenching the fluorescence of anionic chromophore by inhibiting the proton transfer. The influence of ionic denaturants on spectroscopic characteristics of sfGFP complicates the analysis of unfolding – refolding curves. First of all, as visible absorption of sfGFP changes significantly with the change of environment, all recorded values of fluorescence must be corrected for the effect of primary inner filter effect. We proposed a normalization procedure which helps to linearize the dependences of anionic chromophore fluorescence excited at its absorption band at the region of pre-denaturing concentrations of ionic denaturants. When the fluorescence of the chromophore excited at the absorption band of neutral form and recorded at the emission of either neutral or anionic chromophore, the dependences of fluorescence intensity against ionic denaturant concentration are complicated by secondary inner filter effect and inhibition of proton transfer that changes the redistribution of neutral and anionic chromophore after excitation of neutral chromophore. Previously in our studies on GdnHCl-induced denaturation of EGFP, a set of its mutant forms [47] and a series of non-related green and red fluorescent proteins [48], [49] we also have observed the influence of small GdnHCl concentration on chromophore fluorescence. Together with the results obtained in this study we can conclude that potential influence of ionic denaturant on spectroscopic features of FPs should be considered in the study of their folding.

## Supporting Information

File S1
**Figures S1–S3.** Figure S1. Conformational changes in sfGFP induced by GTC. (*A*) Changes in parameter *A =  I*_320_/*I*_365_, λ_ex_ = 297 nm (*B*) Changes in fluorescence anisotropy at an emission wavelength of 365 nm and excitation wavelength of 297 nm (*C*) Changes in the average elution volume of sfGFP calculated as 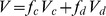, where *f_c_*_(*d*)_ are the proportions of compact (denatured) molecules and *V_c_*_(*d*)_ are elution volumes of molecules in these states. The values of *f_c_*_(*d*)_ are estimated as 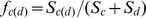, where *S_c_*_(*d*)_ represent the areas under peaks corresponding to compact (denatured) molecules. (*D*) Changes in tryptophan fluorescence intensity recorded at 320 nm. λ_ex_ = 297 nm. (*E* and *F*) Changes in green chromophore fluorescence intensity at two wavelengths of excitation of 365 nm and 470 nm, respectively. Measurements were performed after a 24 h incubation of native protein in the presence of GTC. Figure S2. Ionic denaturant and salt effects on spectral features of sfGFP. The changes in tryptophan fluorescence spectra at an excitation wavelength of 297 nm (first column) and the changes in green fluorescence spectra of sfGFP at excitation wavelengths of 390 nm (second column) and 490 nm (third column). Agents used were ionic denaturants, such as GTC and GdnHCl, and salts, such as NaSCN and NaCl. Applied agent concentrations are indicated on the right of the figure. Figure S3. Conformational changes in sfGFP induced by studied agents. (*A*) Changes in parameter *A =  I*_320_/*I*_365_, λ_ex_ = 297 nm (*B*) Changes in fluorescence anisotropy at an emission wavelength of 365 nm and excitation wavelength of 297 nm. Agents used were GTC (red circles) and GdnHCl (blue circles), NaSCN (pink circles) and NaCl (green circles).(DOC)
