# Supplementary material for: Sensitivity of Superfolder GFP to Ionic Agents
Source: PLoS One. 2014 Oct 27;9(10):e110750. doi: 10.1371/journal.pone.0110750 (PMC4210205; doi:10.1371/journal.pone.0110750)
Supplement: File S1 — Figures S1–S3. Figure S1. Conformational changes in sfGFP induced by GTC. (A) Changes in parameter A = I 320/I 365, λex = 297 nm (B) Changes in fluorescence anisotropy at an emission wavelength of 365 nm and excitation wavelength of 297 nm (C) Changes in the average elution volume of sfGFP calculated as , where fc (d) are the proportions of compact (denatured) molecules and Vc (d) are elution volumes of molecules in these states. The values of fc (d) are estimated as , where Sc (d) represent the areas under peaks corresponding to compact (denatured) molecules. (D) Changes in tryptophan fluorescence intensity recorded at 320 nm. λex = 297 nm. (E and F) Changes in green chromophore fluorescence intensity at two wavelengths of excitation of 365 nm and 470 nm, respectively. Measurements were performed after a 24 h incubation of native protein in the presence of GTC. Figure S2. Ionic denaturant and salt effects on spectral features of sfGFP. The changes in tryptophan fluorescence spectra at an excitation wavelength of 297 nm (first column) and the changes in green fluorescence spectra of sfGFP at excitation wavelengths of 390 nm (second column) and 490 nm (third column). Agents used were ionic denaturants, such as GTC and GdnHCl, and salts, such as NaSCN and NaCl. Applied agent concentrations are indicated on the right of the figure. Figure S3. Conformational changes in sfGFP induced by studied agents. (A) Changes in parameter A = I 320/I 365, λex = 297 nm (B) Changes in fluorescence anisotropy at an emission wavelength of 365 nm and excitation wavelength of 297 nm. Agents used were GTC (red circles) and GdnHCl (blue circles), NaSCN (pink circles) and NaCl (green circles). (DOC) [file pone.0110750.s001.doc]

Supporting information for:

Sensitivity of superfolder GFP to ionic agents*

**Olesya V. Stepanenko^§^, Olga V. Stepanenko^§^, Irina M. Kuznetsova^§,ǂ^, Vladislav V. Verkhusha^¶,#^ and Konstantin K. Turoverov^§,ǂ^**

^§^Laboratory of Structural Dynamics, Stability and Folding of Proteins, Institute of Cytology, Russian Academy of Sciences, St. Petersburg 194064, Russia

^¶^Department of Anatomy and Structural Biology, Albert Einstein College of Medicine, Bronx, New York 10461, USA

^#^Department of Biochemistry and Developmental Biology, Institute of Biomedicine, University of Helsinki, Helsinki 00290, Finland

^ǂ^Department of Biophysics, St. Petersburg State Polytechnical University, St. Petersburg 195251, Russia

To whom correspondence should be addressed: Konstantin K. Turoverov, Laboratory of Structural Dynamics, Stability and Folding of Proteins, Institute of Cytology, Russian Academy of Sciences, St. Petersburg 194064, Russia; Tel: +7 (812) 2971957; Fax: +7 (812) 2970341; E-mail: kkt@incras.ru.

**Keywords**: green fluorescent protein; anion binding; denaturant; salt concentration; cation binding

**Figure S1. Conformational changes in sfGFP induced by GTC.**

(***A***) Changes in parameter *А = I*_320_/*I*_365_, λ_ex_ = 297 nm (***B***) Changes in fluorescence anisotropy at an emission wavelength of 365 nm and excitation wavelength of 297 nm (***C***) Changes in the average elution volume of sfGFP calculated as $V=f_{c}V_{c}+f_{d}V_{d}$, where *f_c_*(*_d_*) are the proportions of compact (denatured) molecules and *V_c_*(*_d_*) are elution volumes of molecules in these states. The values of *f_c_*(*_d_*) are estimated as $f_{c(d)}=S_{c(d)}/(S_{c}+S_{d})$, where *S_c_*(*_d_*) represent the areas under peaks corresponding to compact (denatured) molecules. (***D***) Changes in tryptophan fluorescence intensity recorded at 320 nm. λ_ex_ = 297 nm. (***E*** and ***F***) Changes in green chromophore fluorescence intensity at two wavelengths of excitation of 365 nm and 470 nm, respectively. Measurements were performed after a 24 h incubation of native protein in the presence of GTC.

**Figure S2. Ionic denaturant and salt effects on spectral features of sfGFP.**

The changes in tryptophan fluorescence spectra at an excitation wavelength of 297 nm (first column) and the changes in green fluorescence spectra of sfGFP at excitation wavelengths of 390 nm (second column) and 490 nm (third column). Agents used were ionic denaturants, such as GTC and GdnHCl, and salts, such as NaSCN and NaCl. Applied agent concentrations are indicated on the right of the figure.

**Figure S3. Conformational changes in sfGFP induced by studied agents.**

(***A***) Changes in parameter *А = I*_320_/*I*_365_, λ_ex_ = 297 nm (***B***) Changes in fluorescence anisotropy at an emission wavelength of 365 nm and excitation wavelength of 297 nm. Agents used were GTC (red circles) and GdnHCl (blue circles), NaSCN (pink circles) and NaCl (green circles).
